# Ethyl 6′-amino-5′-cyano-2′-methyl-2-oxospiro­[indoline-3,4′-pyran]-3′-carboxyl­ate

**DOI:** 10.1107/S1600536809054075

**Published:** 2009-12-19

**Authors:** Jing Wang, Song-Lei Zhu

**Affiliations:** a Department of Chemistry, Xuzhou Medical College, Xuzhou 221004, People’s Republic of China

## Abstract

In the title compound, C_17_H_15_N_3_O_4_, the atoms of the spiro pyran ring are nearly planar with a maximum deviation of 0.0188 (14) Å. The benzene and pyrrole rings make a dihedral angle of 5.71 (6)°. The indole system and the pyran ring are oriented at a dihedral angle of 82.94 (3)°. The crystal structure is stabilized by inter­molecular classical and non-classical N—H⋯O, N—H⋯N and C—H⋯O hydrogen bonds.

## Related literature

For the indole nucleus, see: da Silva *et al.* (2001 ▶). For the anti­bacterial and fungicidal activities of indoles, see: Joshi & Chand (1982 ▶). Spiro­oxindole ring systems are found in a number of alkaloids, *e.g.* horsifiline, spiro­tryprostatin and elacomine, see: Abdel-Rahman *et al.* (2004 ▶). For our work on the preparation of heterocyclic compounds involving indole derivatives, see: Zhu *et al.* (2007 ▶).
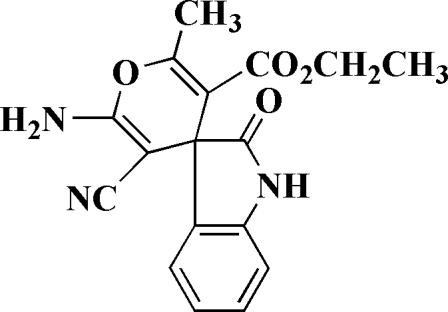


## Experimental

### 

#### Crystal data

C_17_H_15_N_3_O_4_
*M* *_r_* = 325.32
Monoclinic, 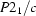

*a* = 7.7812 (16) Å
*b* = 19.998 (4) Å
*c* = 10.044 (2) Å
β = 103.435 (4)°
*V* = 1520.2 (6) Å^3^
*Z* = 4
Mo *K*α radiation
μ = 0.10 mm^−1^
*T* = 153 K
0.60 × 0.30 × 0.24 mm

#### Data collection

Rigaku Mercury diffractometer
Absorption correction: multi-scan (*ABSCOR*; Jacobson, 1998 ▶) *T*_min_ = 0.764, *T*_max_ = 0.975
14692 measured reflections
2779 independent reflections
2550 reflections with *I* > 2σ(*I*)
*R*_int_ = 0.026

#### Refinement

*R*[*F* ^2^ > 2σ(*F* ^2^)] = 0.041
*wR*(*F* ^2^) = 0.096
*S* = 1.14
2779 reflections
228 parameters
H atoms treated by a mixture of independent and constrained refinement
Δρ_max_ = 0.23 e Å^−3^
Δρ_min_ = −0.31 e Å^−3^

### 

Data collection: *CrystalClear* (Rigaku/MSC, 2001 ▶); cell refinement: *CrystalClear*; data reduction: *CrystalStructure* (Rigaku/MSC, 2004 ▶); program(s) used to solve structure: *SHELXS97* (Sheldrick, 2008 ▶); program(s) used to refine structure: *SHELXL97* (Sheldrick, 2008 ▶); molecular graphics: *ORTEPII* (Johnson, 1976 ▶) and *PLATON* (Spek, 2009 ▶); software used to prepare material for publication: *SHELXL97* and *PLATON*.

## Supplementary Material

Crystal structure: contains datablocks global, I. DOI: 10.1107/S1600536809054075/pv2248sup1.cif

Structure factors: contains datablocks I. DOI: 10.1107/S1600536809054075/pv2248Isup2.hkl

Additional supplementary materials:  crystallographic information; 3D view; checkCIF report

## Figures and Tables

**Table 1:** Hydrogen-bond geometry (Å, °)

*D*—H⋯*A*	*D*—H	H⋯*A*	*D*⋯*A*	*D*—H⋯*A*
N1—H1⋯N2^i^	0.88	2.56	3.321 (2)	146
N1—H1⋯O3^ii^	0.88	2.64	3.337 (2)	137
N3—H3*A*⋯N2^iii^	0.88 (2)	2.64 (2)	3.223 (2)	124 (2)
N3—H3*B*⋯O4^iv^	0.91 (2)	1.93 (2)	2.841 (2)	177 (2)
C13—H13⋯O2^v^	0.95	2.50	3.293 (2)	141

Symmetry codes: (i) 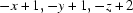; (ii) 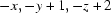; (iii) 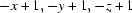; (iv) 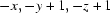; (v) 
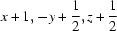
.

**Fig. 1.**
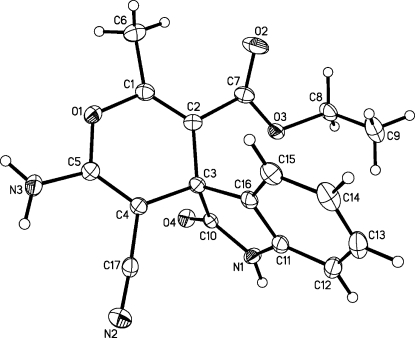
The molecular structure of the title molecule, with the atom-numbering scheme. Displacement ellipsoids are drawn at the 45% probability level.

**Fig. 2.**
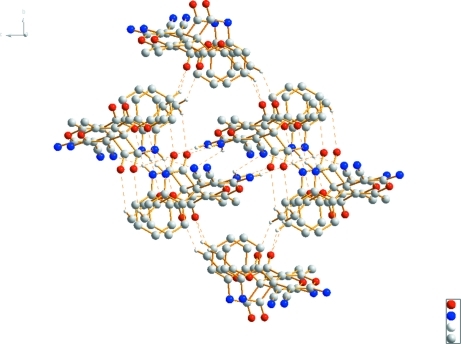
A packing diagram of (I) showing hydrogen bonds as dashed lines.

## supplementary crystallographic information

### Comment

The indole nucleus is a well known heterocycle (da Silva *et al.*, 2001). Compounds containing the indole moiety exhibit antibacterial and fungicidal activities (Joshi & Chand, 1982). Spirooxindole ring systems are found in a number of alkaloids, e.g., horsifiline, spirotryprostatin and elacomine (Abdel-Rahman *et al.*, 2004). As a part of our programme devoted to the preparation of heterocyclic compounds involving indole derivatives (Zhu *et al.*, 2007), we have synthesized a series of spirooxindoles *via* reactions of isatins together with malononitrile and ethyl 3-oxobutanoate in water. We report herein the crystal structure of the title compound, (I).

In the molecule of (I), (Fig. 1), the new formed spiro pyran ring (O1/C1-C5) adopts nearly planar confirmation. Rings (N1/C3/C10/C11/C16) and (C11-C16) of the indole system, are of course planar; the dihedral angle between the mean-planes of the two rings is 5.707 (5)°. The indole system and pyran ring are oriented at a dihedral angle of 82.926 (3)°.

In the crystal structure, intermolecular N—H···O, N—H···N, and C—H···O hydrogen bonds (Table 1) link the molecules (Fig. 2), thus stabilizing the crystal structure.

### Experimental

Compound (I) was prepared by the reaction of isatin (1 mmol), malononitrile (1 mmol) and ethyl 3-oxobutanoate (1 mmol) in water (5 ml). The reaction was catalyzed by TEBAC (triethylbenzylammonium chloride, 1 mmol). After stirring at 333 K for 5 h, the reaction mixture was cooled and washed with a small amount of ethanol. The crude product was filtered and single crystals of the title compound were obtained from ethanol solution by slow evaporation at room temperature (yield; 85%, m.p. 510-511 K).

### Refinement

H atoms (for NH_2_) were located in a difference syntheses and refined. The remaining H atoms were positioned geometrically, with N–H = 0.88 Å and C–H = 0.95 and 0.98 Å for aromatic and methyl H and constrained to ride on their parent atoms with U_iso_(H) = xU_eq_(C,N), where x = 1.5 for methyl and x = 1.2 for all other H atoms.

### Crystal data

**Table:**

C_17_H_15_N_3_O_4_	*F*(000) = 680
*M**_r_* = 325.32	*D*_x_ = 1.421 Mg m^−^^3^
Monoclinic, *P*2_1_/*c*	Melting point = 511–512 K
Hall symbol: -P 2ybc	Mo *K*α radiation, λ = 0.71070 Å
*a* = 7.7812 (16) Å	Cell parameters from 5549 reflections
*b* = 19.998 (4) Å	θ = 3.1–25.3°
*c* = 10.044 (2) Å	µ = 0.10 mm^−^^1^
β = 103.435 (4)°	*T* = 153 K
*V* = 1520.2 (6) Å^3^	Prism, colorless
*Z* = 4	0.60 × 0.30 × 0.24 mm

### Data collection

**Table:**

Rigaku Mercury diffractometer	2779 independent reflections
Radiation source: fine-focus sealed tube	2550 reflections with *I* > 2σ(*I*)
graphite	*R*_int_ = 0.026
Detector resolution: 7.31 pixels mm^-1^	θ_max_ = 25.3°, θ_min_ = 3.2°
ω scans	*h* = −8→9
Absorption correction: multi-scan (*ABSCOR*; Jacobson, 1998)	*k* = −24→24
*T*_min_ = 0.764, *T*_max_ = 0.975	*l* = −12→11
14692 measured reflections	

### Refinement

**Table:**

Refinement on *F*^2^	Primary atom site location: structure-invariant direct methods
Least-squares matrix: full	Secondary atom site location: difference Fourier map
*R*[*F*^2^ > 2σ(*F*^2^)] = 0.041	Hydrogen site location: inferred from neighbouring sites
*wR*(*F*^2^) = 0.096	H atoms treated by a mixture of independent and constrained refinement
*S* = 1.14	*w* = 1/[σ^2^(*F*_o_^2^) + (0.0403*P*)^2^ + 0.642*P*] where *P* = (*F*_o_^2^ + 2*F*_c_^2^)/3
2779 reflections	(Δ/σ)_max_ < 0.001
228 parameters	Δρ_max_ = 0.23 e Å^−^^3^
0 restraints	Δρ_min_ = −0.31 e Å^−^^3^

### Special details

**Table:**

Experimental. Spectroscopic analysis: IR (KBr, n, cm^-1^): 3480, 3372, 3287, 2191, 1721, 1620, 1474, 1381, 1288, 1211, 1072, 756, 679, 625. ^1^H NMR (400 MHz, DMSO-d_6_): 10.39 (s, 1H, NH), 7.13-7.18 (m, 3H, NH_2_ + ArH), 7.03 (d, J = 10.0 Hz, 1H, ArH), 6.90 (t, J = 10.0 Hz, 1H, ArH), 6.77 (d, J = 10.4 Hz, 1H, ArH), 3.71-3.76 (m, 2H, CH_2_), 0.75 (t, J = 9.6 Hz, 3H, CH_3_).
Geometry. All esds (except the esd in the dihedral angle between two l.s. planes) are estimated using the full covariance matrix. The cell esds are taken into account individually in the estimation of esds in distances, angles and torsion angles; correlations between esds in cell parameters are only used when they are defined by crystal symmetry. An approximate (isotropic) treatment of cell esds is used for estimating esds involving l.s. planes.
Refinement. Refinement of F^2^ against ALL reflections. The weighted R-factor wR and goodness of fit S are based on F^2^, conventional R-factors R are based on F, with F set to zero for negative F^2^. The threshold expression of F^2^ > 2sigma(F^2^) is used only for calculating R-factors(gt) etc. and is not relevant to the choice of reflections for refinement. R-factors based on F^2^ are statistically about twice as large as those based on F, and R- factors based on ALL data will be even larger.

### Fractional atomic coordinates and isotropic or equivalent isotropic displacement parameters (Å^2^)

**Table:**

	*x*	*y*	*z*	*U*_iso_*/*U*_eq_	
O1	−0.02630 (14)	0.40611 (6)	0.47320 (10)	0.0250 (3)	
O2	−0.29838 (16)	0.31533 (6)	0.74767 (13)	0.0361 (3)	
O3	−0.15237 (13)	0.38754 (5)	0.90531 (10)	0.0210 (3)	
O4	0.02295 (14)	0.52209 (5)	0.82365 (11)	0.0220 (3)	
N1	0.21452 (16)	0.45913 (6)	0.98355 (12)	0.0198 (3)	
H1	0.2470	0.4898	1.0472	0.024*	
N2	0.52683 (18)	0.47764 (7)	0.73427 (15)	0.0310 (3)	
N3	0.2201 (2)	0.44309 (8)	0.42427 (14)	0.0266 (3)	
H3A	0.322 (3)	0.4642 (10)	0.446 (2)	0.039 (6)*	
H3B	0.144 (3)	0.4529 (10)	0.343 (2)	0.039 (5)*	
C1	−0.1291 (2)	0.38438 (8)	0.56013 (15)	0.0213 (3)	
C2	−0.07153 (19)	0.38461 (7)	0.69607 (15)	0.0186 (3)	
C3	0.11176 (19)	0.40821 (7)	0.76868 (15)	0.0172 (3)	
C4	0.21368 (19)	0.42787 (7)	0.66251 (15)	0.0178 (3)	
C5	0.1420 (2)	0.42671 (7)	0.52598 (15)	0.0198 (3)	
C6	−0.3046 (2)	0.36409 (10)	0.47479 (18)	0.0324 (4)	
H6A	−0.2958	0.3193	0.4374	0.049*	
H6B	−0.3417	0.3960	0.3995	0.049*	
H6C	−0.3916	0.3637	0.5313	0.049*	
C7	−0.18809 (19)	0.35822 (8)	0.78223 (16)	0.0212 (3)	
C8	−0.2410 (2)	0.35970 (8)	1.00633 (16)	0.0252 (4)	
H8A	−0.3478	0.3347	0.9593	0.030*	
H8B	−0.2782	0.3963	1.0598	0.030*	
C9	−0.1167 (3)	0.31383 (10)	1.1002 (2)	0.0395 (5)	
H9A	−0.0801	0.2778	1.0467	0.059*	
H9B	−0.1762	0.2947	1.1673	0.059*	
H9C	−0.0125	0.3390	1.1479	0.059*	
C10	0.10541 (19)	0.47069 (7)	0.86000 (15)	0.0167 (3)	
C11	0.26981 (19)	0.39209 (8)	0.99810 (15)	0.0200 (3)	
C12	0.3617 (2)	0.35940 (9)	1.11389 (17)	0.0267 (4)	
H12	0.4029	0.3825	1.1979	0.032*	
C13	0.3917 (2)	0.29121 (9)	1.10268 (18)	0.0312 (4)	
H13	0.4564	0.2675	1.1802	0.037*	
C14	0.3292 (2)	0.25720 (9)	0.98083 (19)	0.0311 (4)	
H14	0.3489	0.2105	0.9765	0.037*	
C15	0.2374 (2)	0.29119 (8)	0.86432 (17)	0.0249 (4)	
H15	0.1948	0.2681	0.7805	0.030*	
C16	0.21024 (19)	0.35887 (7)	0.87391 (15)	0.0185 (3)	
C17	0.3870 (2)	0.45422 (8)	0.70497 (15)	0.0203 (3)	

### Atomic displacement parameters (Å^2^)

**Table:**

	*U*^11^	*U*^22^	*U*^33^	*U*^12^	*U*^13^	*U*^23^
O1	0.0210 (6)	0.0353 (6)	0.0174 (5)	−0.0048 (5)	0.0020 (4)	0.0002 (5)
O2	0.0352 (7)	0.0357 (7)	0.0425 (7)	−0.0195 (6)	0.0193 (6)	−0.0165 (6)
O3	0.0192 (6)	0.0251 (6)	0.0203 (5)	−0.0058 (4)	0.0075 (4)	−0.0010 (4)
O4	0.0238 (6)	0.0198 (6)	0.0225 (6)	0.0026 (4)	0.0055 (5)	0.0002 (4)
N1	0.0194 (7)	0.0230 (7)	0.0161 (6)	−0.0020 (5)	0.0021 (5)	−0.0028 (5)
N2	0.0216 (8)	0.0377 (8)	0.0339 (8)	−0.0051 (6)	0.0069 (6)	0.0020 (6)
N3	0.0248 (8)	0.0378 (8)	0.0178 (7)	−0.0024 (7)	0.0058 (6)	0.0023 (6)
C1	0.0175 (8)	0.0226 (8)	0.0238 (8)	−0.0010 (6)	0.0049 (6)	−0.0013 (6)
C2	0.0166 (7)	0.0177 (7)	0.0214 (8)	0.0002 (6)	0.0041 (6)	−0.0027 (6)
C3	0.0150 (7)	0.0187 (7)	0.0183 (7)	−0.0005 (6)	0.0050 (6)	0.0001 (6)
C4	0.0163 (7)	0.0191 (7)	0.0185 (7)	0.0008 (6)	0.0050 (6)	−0.0002 (6)
C5	0.0185 (8)	0.0207 (8)	0.0202 (8)	0.0017 (6)	0.0043 (6)	−0.0003 (6)
C6	0.0252 (9)	0.0403 (10)	0.0281 (9)	−0.0073 (8)	−0.0010 (7)	−0.0021 (8)
C7	0.0167 (7)	0.0200 (8)	0.0277 (8)	−0.0009 (6)	0.0069 (6)	−0.0032 (6)
C8	0.0247 (8)	0.0288 (9)	0.0264 (8)	−0.0073 (7)	0.0144 (7)	−0.0009 (7)
C9	0.0426 (11)	0.0362 (10)	0.0437 (11)	−0.0027 (8)	0.0185 (9)	0.0142 (8)
C10	0.0147 (7)	0.0199 (8)	0.0166 (7)	−0.0027 (6)	0.0061 (6)	0.0003 (6)
C11	0.0143 (7)	0.0267 (8)	0.0203 (8)	0.0002 (6)	0.0070 (6)	0.0033 (6)
C12	0.0173 (8)	0.0415 (10)	0.0215 (8)	0.0019 (7)	0.0052 (6)	0.0086 (7)
C13	0.0195 (8)	0.0427 (10)	0.0339 (10)	0.0086 (7)	0.0111 (7)	0.0188 (8)
C14	0.0245 (9)	0.0264 (9)	0.0465 (11)	0.0098 (7)	0.0163 (8)	0.0131 (8)
C15	0.0212 (8)	0.0245 (8)	0.0319 (9)	0.0021 (6)	0.0121 (7)	0.0011 (7)
C16	0.0138 (7)	0.0227 (8)	0.0204 (8)	−0.0002 (6)	0.0072 (6)	0.0024 (6)
C17	0.0209 (8)	0.0242 (8)	0.0167 (7)	0.0022 (6)	0.0059 (6)	0.0024 (6)

### Geometric parameters (Å, °)

**Table:**

O1—C5	1.3577 (19)	C4—C17	1.418 (2)
O1—C1	1.3840 (19)	C6—H6A	0.9800
O2—C7	1.2058 (19)	C6—H6B	0.9800
O3—C7	1.3378 (19)	C6—H6C	0.9800
O3—C8	1.4631 (18)	C8—C9	1.497 (3)
O4—C10	1.2218 (18)	C8—H8A	0.9900
N1—C10	1.3508 (19)	C8—H8B	0.9900
N1—C11	1.405 (2)	C9—H9A	0.9800
N1—H1	0.8800	C9—H9B	0.9800
N2—C17	1.158 (2)	C9—H9C	0.9800
N3—C5	1.345 (2)	C11—C12	1.380 (2)
N3—H3A	0.88 (2)	C11—C16	1.394 (2)
N3—H3B	0.91 (2)	C12—C13	1.392 (3)
C1—C2	1.334 (2)	C12—H12	0.9500
C1—C6	1.490 (2)	C13—C14	1.386 (3)
C2—C7	1.488 (2)	C13—H13	0.9500
C2—C3	1.518 (2)	C14—C15	1.397 (2)
C3—C16	1.517 (2)	C14—H14	0.9500
C3—C4	1.521 (2)	C15—C16	1.377 (2)
C3—C10	1.558 (2)	C15—H15	0.9500
C4—C5	1.355 (2)		
			
C5—O1—C1	119.69 (11)	O3—C8—C9	109.26 (13)
C7—O3—C8	116.35 (12)	O3—C8—H8A	109.8
C10—N1—C11	111.76 (12)	C9—C8—H8A	109.8
C10—N1—H1	124.1	O3—C8—H8B	109.8
C11—N1—H1	124.1	C9—C8—H8B	109.8
C5—N3—H3A	118.2 (13)	H8A—C8—H8B	108.3
C5—N3—H3B	114.7 (13)	C8—C9—H9A	109.5
H3A—N3—H3B	118.5 (19)	C8—C9—H9B	109.5
C2—C1—O1	122.60 (13)	H9A—C9—H9B	109.5
C2—C1—C6	129.37 (14)	C8—C9—H9C	109.5
O1—C1—C6	108.01 (13)	H9A—C9—H9C	109.5
C1—C2—C7	119.26 (14)	H9B—C9—H9C	109.5
C1—C2—C3	123.16 (13)	O4—C10—N1	126.37 (14)
C7—C2—C3	117.53 (13)	O4—C10—C3	125.82 (13)
C16—C3—C2	113.49 (12)	N1—C10—C3	107.73 (12)
C16—C3—C4	113.29 (12)	C12—C11—C16	121.85 (15)
C2—C3—C4	109.16 (12)	C12—C11—N1	128.74 (15)
C16—C3—C10	101.03 (12)	C16—C11—N1	109.36 (13)
C2—C3—C10	112.18 (12)	C11—C12—C13	117.36 (16)
C4—C3—C10	107.37 (11)	C11—C12—H12	121.3
C5—C4—C17	116.72 (13)	C13—C12—H12	121.3
C5—C4—C3	123.09 (13)	C14—C13—C12	121.40 (15)
C17—C4—C3	119.97 (13)	C14—C13—H13	119.3
N3—C5—C4	127.76 (14)	C12—C13—H13	119.3
N3—C5—O1	110.01 (13)	C13—C14—C15	120.47 (16)
C4—C5—O1	122.21 (13)	C13—C14—H14	119.8
C1—C6—H6A	109.5	C15—C14—H14	119.8
C1—C6—H6B	109.5	C16—C15—C14	118.46 (16)
H6A—C6—H6B	109.5	C16—C15—H15	120.8
C1—C6—H6C	109.5	C14—C15—H15	120.8
H6A—C6—H6C	109.5	C15—C16—C11	120.43 (14)
H6B—C6—H6C	109.5	C15—C16—C3	130.66 (14)
O2—C7—O3	123.98 (14)	C11—C16—C3	108.82 (13)
O2—C7—C2	125.02 (14)	N2—C17—C4	176.79 (16)
O3—C7—C2	110.98 (12)		
			
C5—O1—C1—C2	2.1 (2)	C3—C2—C7—O3	30.15 (18)
C5—O1—C1—C6	−179.13 (13)	C7—O3—C8—C9	98.97 (16)
O1—C1—C2—C7	−177.92 (13)	C11—N1—C10—O4	172.10 (14)
C6—C1—C2—C7	3.6 (3)	C11—N1—C10—C3	−11.17 (16)
O1—C1—C2—C3	−0.4 (2)	C16—C3—C10—O4	−172.22 (14)
C6—C1—C2—C3	−178.80 (15)	C2—C3—C10—O4	−51.02 (19)
C1—C2—C3—C16	−129.44 (15)	C4—C3—C10—O4	68.90 (18)
C7—C2—C3—C16	48.16 (17)	C16—C3—C10—N1	11.03 (14)
C1—C2—C3—C4	−2.1 (2)	C2—C3—C10—N1	132.23 (13)
C7—C2—C3—C4	175.55 (12)	C4—C3—C10—N1	−107.85 (13)
C1—C2—C3—C10	116.82 (16)	C10—N1—C11—C12	−170.85 (15)
C7—C2—C3—C10	−65.58 (16)	C10—N1—C11—C16	6.45 (17)
C16—C3—C4—C5	130.57 (15)	C16—C11—C12—C13	−0.6 (2)
C2—C3—C4—C5	3.1 (2)	N1—C11—C12—C13	176.36 (14)
C10—C3—C4—C5	−118.77 (15)	C11—C12—C13—C14	−1.1 (2)
C16—C3—C4—C17	−54.99 (18)	C12—C13—C14—C15	1.6 (2)
C2—C3—C4—C17	177.50 (13)	C13—C14—C15—C16	−0.3 (2)
C10—C3—C4—C17	55.67 (17)	C14—C15—C16—C11	−1.4 (2)
C17—C4—C5—N3	5.3 (2)	C14—C15—C16—C3	−177.64 (14)
C3—C4—C5—N3	179.89 (15)	C12—C11—C16—C15	1.9 (2)
C17—C4—C5—O1	−176.31 (13)	N1—C11—C16—C15	−175.60 (13)
C3—C4—C5—O1	−1.7 (2)	C12—C11—C16—C3	178.90 (13)
C1—O1—C5—N3	177.58 (13)	N1—C11—C16—C3	1.38 (16)
C1—O1—C5—C4	−1.1 (2)	C2—C3—C16—C15	49.0 (2)
C8—O3—C7—O2	6.8 (2)	C4—C3—C16—C15	−76.2 (2)
C8—O3—C7—C2	−172.00 (12)	C10—C3—C16—C15	169.28 (15)
C1—C2—C7—O2	29.0 (2)	C2—C3—C16—C11	−127.57 (13)
C3—C2—C7—O2	−148.67 (16)	C4—C3—C16—C11	107.22 (14)
C1—C2—C7—O3	−152.16 (14)	C10—C3—C16—C11	−7.30 (15)

### Hydrogen-bond geometry (Å, °)

**Table:**

*D*—H···*A*	*D*—H	H···*A*	*D*···*A*	*D*—H···*A*
N1—H1···N2^i^	0.88	2.56	3.321 (2)	146
N1—H1···O3^ii^	0.88	2.64	3.337 (2)	137
N3—H3A···N2^iii^	0.88 (2)	2.64 (2)	3.223 (2)	124 (2)
N3—H3B···O4^iv^	0.91 (2)	1.93 (2)	2.841 (2)	177 (2)
C13—H13···O2^v^	0.95	2.50	3.293 (2)	141

Symmetry codes: (i) −*x*+1, −*y*+1, −*z*+2; (ii) −*x*, −*y*+1, −*z*+2; (iii) −*x*+1, −*y*+1, −*z*+1; (iv) −*x*, −*y*+1, −*z*+1; (v) *x*+1, −*y*+1/2, *z*+1/2.

## References

[bb1] Abdel-Rahman, A. H., Keshk, E. M., Hanna, M. A. & El-Bady, Sh. M. (2004). *Bioorg. Med. Chem.* **12**, 2483–2488. 10.1016/j.bmc.2003.10.063 15080944

[bb2] Jacobson, R. (1998). *ABSCOR* Private communication to the Rigaku Corporation, Tokyo, Japan.

[bb3] Johnson, C. K. (1976). *ORTEPII* Report ORNL-5138. Oak Ridge National Laboratory, Tennessee, USA.

[bb4] Joshi, K. C. & Chand, P. (1982). *Pharmazie*, **37**, 1–12. 10.1002/chin.198221355 7041138

[bb5] Rigaku/MSC (2001). *CrystalClear* Rigaku/MSC, The Woodlands, Texas, USA.

[bb6] Rigaku/MSC (2004). *CrystalStructure* Rigaku/MSC, The Woodlands, Texas, USA.

[bb7] Sheldrick, G. M. (2008). *Acta Cryst.* A**64**, 112–122. 10.1107/S0108767307043930 18156677

[bb8] Silva, J. F. M. da, Garden, S. J. & Pinto, A. C. (2001). *J. Braz. Chem. Soc.* **12**, 273–324.

[bb9] Spek, A. L. (2009). *Acta Cryst.* D**65**, 148–155. 10.1107/S090744490804362X PMC2631630 19171970

[bb10] Zhu, S. L., Ji, S. J. & Zhang, Y. (2007). *Tetrahedron*, **63**, 9365–9372.

